# Calories From Beverages Purchased at 2 Major Coffee Chains in New York City, 2007

**Published:** 2009-09-15

**Authors:** Christina Huang, Tamara Dumanovsky, Lynn D. Silver, Cathy Nonas, Mary T. Bassett

**Affiliations:** New York City Department of Health and Mental Hygiene; New York City Department of Health and Mental Hygiene, New York, New York; New York City Department of Health and Mental Hygiene, New York, New York; New York City Department of Health and Mental Hygiene, New York, New York; Doris Duke Charitable Foundation, New York, New York. At the time of this research, Dr Bassett was affiliated with the New York City Department of Health and Mental Hygiene, New York, New York

## Abstract

**Introduction:**

Calorie intake from beverages has increased in the past decades, which most likely contributes to higher obesity rates. Although coffee chains have grown in popularity in recent years, few data examine the calorie contribution of these drinks. We examined afternoon beverage purchases in New York City at 2 major coffee chains and estimated the mean calorie content of these beverages.

**Methods:**

We collected purchase receipts and brief surveys from adult customers at 42 Starbucks and 73 Dunkin' Donuts stores during the spring of 2007. For each purchase, we obtained the calorie content from the company's Web site; these values were adjusted to account for self-reported customization of the drink.

**Results:**

We included 1,127 beverage purchases at Starbucks and 1,830 at Dunkin' Donuts in our analyses. Brewed coffee or tea averaged 63 kcal, and blended coffee beverages averaged 239 kcal. Approximately two-thirds of purchases at Starbucks and one-fourth of purchases at Dunkin' Donuts were blended coffee beverages.

**Conclusion:**

Calories in blended coffee beverages are high; on average, customers bought 12% of a 2,000-kcal diet. Policy changes to provide for calorie posting at the point of purchase could increase customer awareness of the calories in these beverages; modifying standard formulations of blended coffee beverages, such as using low-fat milk or smaller serving sizes, would also reduce calorie content.

## Introduction

Coffee chains and specialty coffee outlets have proliferated in recent years. Starbucks, the largest coffee chain in the world, had more than 11,000 retail stores in the United States and 15,000 stores worldwide as of December 2008; Dunkin' Donuts had more than 5,700 stores in the United States and nearly 8,000 stores worldwide at the end of 2007. Both companies have promoted their products aggressively and serve millions of customers each day. In 2007, the United States National Coffee Association found that more than half (55%) of the adult population drinks coffee daily (1). "Blended coffee beverages," a term we use to describe any drink, excluding brewed coffees and teas, prepared at the counter of a coffee retailer, have increased in popularity; 17% of adults report drinking them daily (2). The calorie contribution of these blended coffee beverages, marketed as afternoon "pick-me-ups," has attracted little attention in nutritional research, which has focused instead on caffeine and other active compounds in coffee (3) and on the calorie content of brewed coffee or tea (4).

Blended coffee beverages have many more calories than does a brewed cup of coffee or tea, to which calories are introduced mainly from added milk or sugar. One high-calorie blended coffee beverage sold at Starbucks is the Strawberries & Crème Frappuccino Blended Crème; the largest size ("venti," 24 oz) with whipped cream contains 750 kcal, or approximately 38% of the 2,000-kcal diet often used as a benchmark for total daily calorie intake. A large Dunkin' Donuts Vanilla Bean Coolatta (32 oz) contains 860 kcal.

Daily calorie intake from beverages has increased in the past several decades in the United States. One study found that overall daily calorie intake from beverages increased by 222 kcal per person from 1965 to 2002; this increase resulted largely from drinking sweetened beverages (5). Another study found that daily calorie intake from sweetened beverages increased from 70 to 190 kcal per person from 1977 to 2001 (4). These trends, combined with the increasing prevalence of obesity in the United States (6,7), indicate that the effect of beverages from popular coffee chains merits examination. We used customers' receipts to estimate calorie values for afternoon beverage purchases among customers at a sample of Starbucks and Dunkin' Donuts stores in New York City.

## Methods

### Survey

In the spring of 2007, the New York City Department of Health and Mental Hygiene (DOHMH) surveyed customers at fast food and coffee chains as part of an evaluation of a new calorie labeling regulation; additional details are provided elsewhere (8). A computer-generated random sample of 300 fast food and coffee chains was selected from approximately 1,625 locations in New York City. The data presented here are limited to the 2 coffee chains included in this larger study: 42 Starbucks stores out of a total of 218 citywide and 73 Dunkin' Donuts stores out of a total of 343 citywide.

Data were collected at Starbucks and Dunkin' Donuts locations on weekdays from 2:00 to 4:00 pm for 11 weeks between March and June 2007. Three-person data-collection teams stationed in front of the sampled locations asked customers as they entered the store to save their register receipts. On exiting, customers aged 18 years or older were invited to answer a brief survey and provide their receipts. A $2.00 New York City MetroCard (a public transportation pass good for 1 subway or bus ride) was offered as an incentive for participation.

In addition to providing receipts, customers answered a series of questions to clarify their purchases and supplement the information on the receipts. The brief survey asked customers whether they made any modifications to their beverages, such as adding sugar, milk, or whipped cream. If applicable, data collectors inquired about the type of milk (whole, 2%, skim, soy, cream) and sweetener (sugar or zero-calorie sugar substitute). No personal identifying information was collected, and surveys were collected in English only. The study protocol was submitted to the DOHMH institutional review board and determined to be exempt.

### Data entry

Receipt and survey data were entered into a relational database. Each item was entered as it appeared on the receipt. Additional information about the purchase obtained from the customer survey was also entered (eg, extra items, order modifications).

Beverages were grouped into broad categories on the basis of the classification used in Starbucks and Dunkin' Donuts nutrition guides posted on their Web sites and menus. The beverage categories were brewed coffee and tea and blended coffee beverages, which included presweetened (iced coffee, iced tea, and tea lemonade), milk-based (café au lait, cappuccino, latte, crème, hot chocolate, macchiato, mocha), ice-blended (Frappuccino, fruit juice blend, Coolatta, smoothie), and premixed (chai, Dunkachino, hot chocolate, Turbo) beverages prepared at the counter. Modifications such as adding extra milk, sugar, syrup, or whipped cream did not change the beverage classification.

We used calorie information posted on the Starbucks and Dunkin' Donuts Web sites as of March 1, 2007, to ascribe a calorie count to each beverage. These data were further adjusted on the basis of customer report of extras and modifications for which calorie information was available. For beverages made with milk, if the customer did not know what type of milk was used (approximately half of the customers), we used whole milk (the standard milk used by the chains at the time of the study) to determine calorie counts. For brewed coffee and tea orders with added milk or sugar, calories were adjusted proportionately by drink size. Calories for 1 oz of milk of the type specified by the customer or two 2.8-g packets of sugar (22 kcal) were added per 12 oz at Starbucks and per 10 oz at Dunkin' Donuts (standard small beverages).

### Statistical analysis

All analyses were completed in SPSS version 15.0 (SPSS, Inc, Chicago, Illinois). We used the SPSS complex samples module to adjust for the clustered sample design (customers at locations) when calculating mean calorie values and standard errors.

## Results

We excluded 5 of the 42 sampled Starbucks and 3 of the 73 sampled Dunkin' Donuts stores because they were located in less-accessible places (such as an airport or mall) (n = 5), had closed (n = 2), or had uncooperative management (n = 1). We collected 1,285 Starbucks receipts and 2,753 Dunkin' Donuts receipts, which accounted for 42% of eligible customers who entered the store during data collection hours. This participation rate includes high-volume stores where data collectors could not approach all customers. We excluded 53 (4%) Starbucks receipts and 343 (13%) Dunkin' Donuts receipts because the receipt included purchases for someone other than the participant, listed 1 or more items with indeterminate calorie information, came from a nonsampled chain (such as Baskin Robbins), or contained only items not for consumption (such as gift card or coffee mug). Analyses were limited to beverage calories from receipts that included a beverage prepared at the counter, such as coffee, tea, hot chocolate, lattes, and ice-blended and premixed drinks; bottled drinks were excluded. After exclusions, the final data set included 1,127 Starbucks receipts and 1,830 Dunkin' Donuts receipts.

In general, brewed coffee and tea were more popular than blended coffee beverages; however, the popularity varied by coffee chain (Table 1). More Starbucks customers bought blended coffee beverages, and more Dunkin' Donuts customers bought brewed coffee and tea. On average, beverage purchases at Starbucks (156 kcal) were more caloric than those at Dunkin' Donuts (117 kcal). Although the types of purchases differed between the 2 chains, the calorie values for the 2 categories of beverages did not. Brewed coffee or tea averaged 63 kcal, but blended coffee beverages had almost 4 times as many calories, 239 kcal. Of the blended coffee beverages that were purchased, 30% were more than 300 kcal, the amount of a snack or small meal. The average blended coffee beverage accounted for approximately 12% of a 2,000-kcal diet.

### Starbucks

One-third of all beverage purchases at Starbucks were for brewed coffee or tea (Table 2). Of the 377 customers who purchased brewed coffee or tea, almost 75% added milk or sugar. Half cream/half milk and whole milk were popular additions. Even minor modifications increased calories, and the mean calorie value of brewed coffee and tea purchases was estimated to be 38 kcal.

Two-thirds of Starbucks customers ordered a blended coffee beverage. Of blended beverages, presweetened drinks, such as iced teas and iced coffees, had the lowest mean calorie value (121 kcal). Milk-based drinks, such as lattes, were the most popular blended coffee beverage at Starbucks; the calorie content varied depending upon the type of milk used, but more than half of these customers purchased more than 200 kcal in a single drink. Most of the milk-based drinks were made with whole milk. On average, the customers who chose nonfat milk purchased 76 fewer calories than did customers who chose whole milk and 32 fewer calories than customers who chose soy milk.

At Starbucks, the category with the most calories (mean = 306) was the ice-blended beverage group, which represented 22% of blended coffee beverage purchases. Only 20% of these customers chose the "light" option, a lower-calorie version of the same flavor drink with half the average calories. Forty percent opted for whipped cream, which added 60 to 150 kcal to their beverages, depending on the drink type and size. More than half of these drinks (55%) were more than 300 calories.

### Dunkin' Donuts

Most drinks (78%) purchased at Dunkin' Donuts were brewed coffees or teas. Most customers added both milk and sugar to their coffee or tea (55%); only 10% added neither (Table 3). Although whole milk and half cream/half milk were popular modifications, the brewed coffee and tea purchases were the least caloric, averaging an estimated 69 kcal per drink.

Less than one-fourth of Dunkin' Donuts customers (22%) ordered a blended coffee beverage. At the time of the survey, Dunkin' Donuts offered little variety in blended coffee beverages. Milk-based blended coffees, a category limited to cappuccinos and lattes, averaged 162 kcal. Most of these drinks were made with whole milk (85%), which added an extra 75 kcal on average than did nonfat milk.

Approximately 30% of customers who purchased a blended coffee beverage chose a premixed beverage, which did not allow for customer modifications of milk type or sweetener. Despite customer preference for smaller sizes, premixed beverages averaged 265 kcal each. Almost 90% of the premixed drinks were more than 200 kcal.

The most popular type of blended coffee beverage, ice-blended, also had the most calories. Although most customers ordered the small size, these drinks came in larger portions than did beverages that were served hot. All of the ice-blended beverages ordered were more than 200 kcal, and 72% were more than 300 kcal.

## Discussion

The average calorie content of a beverage purchased at Starbucks and Dunkin' Donuts is similar to that of a standard 12-oz can of sugar-sweetened soda. At both chains, ice-blended drinks had the highest calorie content — at more than 300 kcal on average, these drinks have calories equivalent to a scoop of high-fat ice cream. Furthermore, the popularity of high-calorie blended coffee beverages could signify an alarming trend for a new category of sugar-sweetened beverages. Almost 60% of customers who ordered a blended coffee beverage purchased more than 10% of their calories for the day in a single drink purchase, given a 2,000-kcal/d diet. If uncompensated, an extra 200 kcal each day would translate to weight gain of 20 lbs in 1 year.

Because these data were collected from 2:00 to 4:00 pm, many customers probably bought these high-calorie beverages as afternoon "pick-me-ups," in addition to their lunchtime meals. A study of college women found that participants consumed more calories and sugar on days they drank blended coffee beverages, which suggests that people do not compensate for the extra calories by eating less at subsequent meals (9). In addition, research has shown that calories consumed as liquids result in greater overall daily energy intake (10), beverages drunk at breakfast have no effect on calorie intake at lunch (11), and energy intake from food during a single meal is the same, regardless of energy intake from beverages (12). Finally, in the absence of calorie information available at the point of purchase, customers most likely have little idea of the number of calories in these beverages.

This study has some inherent limitations. First, these data reflect calories purchased, not consumed. Nonetheless, research suggests that people's consumption depends on the amount of food presented to them (13-15). Thus, customers probably drank most if not all of the beverage purchased, regardless of size. Second, customers who agreed to participate may have differed from those who refused. However, the proportion of customers who participated varied primarily by customer volume, which suggests that individual factors were not major determinants of participation rates. Additionally, the study targeted purchases from 2:00 to 4:00 pm. People who frequent these chains in the afternoon may be different from those who visit during other times of the day. Seasonality may also have been a factor; because data were collected in the spring, we do not know if similar purchasing patterns hold year-round. Finally, because reliable measurement estimates from customers could not be collected, calorie values for added milk and sugar were standardized to 1 oz of milk and 2 packets of sugar (per 12 oz at Starbucks and per 10 oz at Dunkin' Donuts). Since anecdotally, coffee drinkers add more sugar and milk on average, these results are most likely conservative calorie estimates and underestimate actual calories purchased.

Proposed guidelines for beverage consumption suggest that 50% of daily fluid intake comes from water and 29% from unsweetened tea or coffee (16). As quantified here, coffee chain beverages can range from fewer than 10 kcal in black brewed coffee or tea to as high as 750 kcal in large ice-blended beverages. Given the popularity of blended coffee beverages and the likelihood that consumers do not realize their high calorie content, consumer awareness should be increased, and the industry should be encouraged to provide and promote less-caloric alternatives. Menu labeling has been suggested as a possible solution. A study focusing on a sandwich chain that had calorie information posted prominently at the point of purchase found that customers who reported seeing calorie information inside the restaurant bought fewer calories than those who did not report seeing the information (8). Calorie labeling could encourage customers to add blended coffee beverages, along with soda and candy, to their list of high-calorie products.

Small changes on the industry's part could also help reduce calorie intake. The high calorie content of blended coffee beverages is attributable in part to the large portion sizes. At Dunkin' Donuts, the sizing for small, medium, and large ice-blended drinks is 16 oz, 24 oz, and 32 oz, respectively, and the average calorie content we calculated was 397 kcal. However, if Dunkin' Donuts adopted Starbucks sizing of 12 oz, 16 oz, and 24 oz for its ice-blended beverages, the average calories in beverages offered would drop to 285 kcal. Similarly, if customers who purchased large ice-blended beverages from Starbucks had ordered the medium size instead, their calorie intake would be reduced by 80 kcal (from 420 kcal to 340 kcal). At the time of our study, Starbucks' smallest beverage was 12 oz; however, Starbucks has recently reintroduced an 8-oz size ("short") that would allow customers to get their favorite drinks for one-third fewer calories. Portion sizes greatly influence the amount of food consumed (17-19), which suggests that if coffee chains served their beverages in smaller containers, customers could drink less but still be satisfied (20).

Another way that coffee chains could reduce calories is by changing the standard formulations of their beverages. The soda industry responded to calorie concerns of its customers by introducing diet sodas in the 1980s; in the past year, the sports drink industry has also introduced less-caloric drinks. Recent changes at Starbucks suggest that the company may be responding to similar consumer concerns. In 2007, Starbucks announced a switch from whole milk to 2% milk as the standard formulation for milk-based beverages (21), which translates to 20 to 50 fewer calories per drink. Starbucks has also recently promoted its "light" options, including the Skinny Latte, a latte made with lower-calorie substitutions. In addition, its new line of breakfast items is focused on providing healthy options. Similarly, Dunkin' Donuts has been aggressively marketing its new DDSMART menu, advertised as "Better-For-You Choices" that include lower-calorie breakfast foods and beverages made with skim milk and zero-calorie sweeteners.

In conclusion, blended coffee beverages served in popular coffee chains are high in calories and most likely contribute to the obesity epidemic. Policy changes that require calories to be posted at the point of purchase could increase customer awareness of the calories in these drinks. Reducing portion sizes, offering healthier options, and changing the fat content of standard formulations could also reduce calorie intake. These simple modifications could decrease calorie intake from drinks and help curb the obesity epidemic.

## Acknowledgments

This research was funded through the New York City DOHMH. We thank our colleagues at the New York City DOHMH for their valuable assistance: Thomas Matte for designing the study, Thomas Farley and Thomas R. Frieden for thoughtful comments on earlier drafts, and Rosalyne Tu for data analyses related to the study. We also thank Domingo Pinero and Kristie Lancaster of the Department of Nutrition, Food Studies, and Public Health at New York University for their role in data collection and Mary Northridge of the Mailman School of Public Health at Columbia University for her editorial comments. Both New York University and Dr Northridge received compensation for their work.

## Figures and Tables

**Table 1:** Beverage Purchases and Mean Calories per Customer, Starbucks and Dunkin' Donuts, New York City, 2007

Type of Beverage	n (%)	Mean (SD) Calories, kcal	% of Purchases in Calorie Ranges, kcal^a^				

			<100	100-199	200-299	300-399	≥400
**Both chains**	2,957 (100)	132 (184)	56	21	11	8	4
Brewed coffee/tea	1,799 (61)	63 (57)	87	13	0	0	0
Blended beverage	1,158 (39)	239 (167)	9	33	29	20	10
**Starbucks**	1,127 (100)	156 (153)	40	27	19	10	5
Brewed coffee/tea	377 (34)	38 (30)	97	3	0	0	0
Blended beverage	750 (66)	215 (115)	10	39	28	14	8
**Dunkin' Donuts**	1,830 (100)	117 (149)	67	17	7	6	3
Brewed coffee/tea	1,422 (78)	69 (36)	84	16	0	0	0
Blended beverage	408 (22)	284 (131)	5	23	30	29	14

Abbreviation: SD, standard deviation.

a Percentages may not total 100 because of rounding.

**Table 2:** Starbucks Beverage Purchases and Mean Calories, New York City, 2007

Type of Beverage	n (%)^a^	Mean (SD) Calories, kcal	Size of Beverage, %^a^		

			Small/Tall (12 oz)	Medium (16 oz)	Large (20 oz hot, 24 oz cold)
**Brewed coffee/tea**	377 (100)	38 (30)	64	26	10
Black	101 (27)	13 (30)	71	19	10
Sugar added^b^	14 (4)	37 (21)	71	7	21
Milk added^c^	141 (37)	36 (13)	66	26	8
Sugar and milk added^b^,^c^	121 (32)	63 (19)	55	33	12
**Blended beverages**	750 (100)	215 (116)	44	40	16
Presweetened	175 (23)	121 (60)	25	44	31
No milk	73 (10)	104 (45)	27	40	33
Milk^b^	102 (14)	132 (49)	23	47	30
Milk-based	406 (54)	217 (110)	47	41	11
Whole milk	230 (31)	246 (109)	52	39	9
Nonfat milk	123 (16)	170 (58)	40	47	13
Soy milk	53 (7)	202 (75)	43	40	17
Ice-blended	169 (22)	306 (114)	54	35	11
Regular	135 (18)	339 (76)	57	33	10
Light	34 (4)	171 (42)	44	44	12
**Total**	1,127	156 (154)	50	36	14

Abbreviation: SD, standard deviation.

a Percentages may not total 100 because of rounding.

b Proportionate addition of 2 sugar packets (22 kcal) per 12-oz beverage.

c Proportionate addition of 1 oz milk per 12-oz beverage.

**Table 3:** Dunkin' Donuts Beverage Purchases and Mean Calories, New York City, 2007

Type of Beverage	n (%)^a^	Mean (SD) Calories, kcal	Size of Beverage, %^a^		

			Small (10 oz hot, 16 oz cold)	Medium (16 oz hot, 24 oz cold)	Large (24 oz hot, 32 oz cold)
**Brewed coffee/tea**	1,422 (100)	69 (36)	44	44	12
Black	141 (10)	21 (9)	40	33	26
Sugar added^b^	53 (4)	40 (14)	68	21	11
Milk added^c^	443 (31)	57 (30)	44	46	10
Sugar and milk added^b^,^c^	785 (55)	87 (29)	44	46	10
**Blended beverages**	408 (100)	284 (131)	49	43	8
Milk-based	129 (32)	162 (73)	41	50	9
Whole milk	110 (27)	173 (66)	43	49	8
Nonfat milk	19 (5)	98 (32)	32	53	16
Ice-blended	158 (39)	397 (111)	59	32	10
Premixed	121 (30)	265 (55)	46	50	5
**Total**	1,830	117 (150)	46	44	11

a Percentages may not total 100 because of rounding.

b Proportionate addition of 2 sugar packets (22 kcal) per 10-oz beverage.

c Proportionate addition of 1 oz milk per 10-oz beverage.
